# First estimates of fine root production in tropical peat swamp and *terra firme* forests of the central Congo Basin

**DOI:** 10.1038/s41598-023-38409-x

**Published:** 2023-07-29

**Authors:** Matteo Sciumbata, Yeto Emmanuel Mampouya Wenina, Mackline Mbemba, Greta C. Dargie, Andy J. Baird, Paul J. Morris, Suspense Averti Ifo, Rien Aerts, Simon L. Lewis

**Affiliations:** 1 Section Systems Ecology, Amsterdam Institute for Life and Environment (A-LIFE), Vrije Universiteit, Amsterdam, The Netherlands; 2 École Normale Supérieure, Departement des sciences et vie de la terre, Université Marien Ngouabi, Brazzaville, Republic of the Congo; 3 School of Geography, University of Leeds, Leeds, UK; 4 Department of Geography, University College London, London, UK

**Keywords:** Ecosystem ecology, Carbon cycle, Wetlands ecology, Plant ecology, Tropical ecology

## Abstract

Tropical peatlands are carbon-dense ecosystems because they accumulate partially-decomposed plant material. A substantial fraction of this organic matter may derive from fine root production (FRP). However, few FRP estimates exist for tropical peatlands, with none from the world’s largest peatland complex in the central Congo Basin. Here we report on FRP using repeat photographs of roots from in situ transparent tubes (minirhizotrons), measured to 1 m depth over three one-month periods (spanning dry to wet seasons), in a palm-dominated peat swamp forest, a hardwood-dominated peat swamp forest, and a *terra firme* forest. We find FRP of 2.6 ± 0.3 Mg C ha^−1^ yr^−1^, 1.9 ± 0.5 Mg C ha^−1^ yr^−1^, and 1.7 ± 0.1 Mg C ha^−1^ yr^−1^ in the three ecosystem types respectively (mean ± standard error; no significant ecosystem type differences). These estimates fall within the published FRP range worldwide. Furthermore, our hardwood peat swamp estimate is similar to the only other FRP study in tropical peatlands, also hardwood-dominated, from Micronesia. We also found that FRP decreased with depth and was the highest during the dry season. Overall, we show that minirhizotrons can be used as a low-disturbance method to estimate FRP in tropical forests and peatlands.

## Introduction

Soil organic matter is the largest terrestrial reservoir of organic carbon on Earth^1, 2^. Most of the soil organic matter is derived from aboveground and belowground plant production via litter fall and roots^3^. Of these two inputs to soil organic matter, roots are more efficiently converted into stable organic compounds due to their biochemical composition and direct contact with soil microbes and mineral surfaces^2–4^. Furthermore, fine roots—typically defined as having a diameter of less than 2 mm^5^—are estimated to account for as much as 30% of annual net primary production^6^, making them a significant source of soil organic carbon^7^. Thus, obtaining accurate fine root production (FRP) measurement is important.

Globally, two major patterns point to the importance of estimating FRP in tropical peatlands. First, FRP is at its highest in the tropics, unsurprisingly, given overall high rates of ecosystem productivity^8–11^. Second, peatlands accumulate partially decayed organic material from dead vegetation, resulting in some of the most carbon dense ecosystems on Earth^12, 13^. Therefore, understanding FRP in these systems is a priority. Yet, FRP estimates are rare in tropical peatlands globally with, to our knowledge, just one estimate of FRP in this ecosystem, from Micronesia, having been published^14^. For the world’s largest tropical peatland complex, in the central Congo Basin^13^, we lack any FRP estimate. The scarcity of FRP estimates in tropical peatlands partly stems from the methodological difficulties of working in these environments, particularly studying belowground processes under waterlogged conditions.

Critically, standard methods for assessing FRP in *terra firme* (non-flooded, non-riverine) tropical forests are unsuitable for tropical peat soils. The application of ingrowth cores (cages filled with root-free soil that capture new root growth) and rhizotrons (vertically cut soil pits fitted with a window to measure fine root growth)^15^ is hampered in tropical peatlands because of dense root mats (removing all roots from peat for ingrowth cores is challenging) and waterlogged conditions (rhizotron windows cannot be seen because they are under water). To overcome these problems for estimating FRP in our study area in the peatlands of the central Congo Basin we used a minirhizotron technique recently developed for mangrove soils—the EnRoot system^16^. The EnRoot minirhizotron system comprises clear acrylic tubes installed in the soil, into which an imaging module can be inserted to take repeated photographs over time to determine rates of change in root length^17^. This allows accurate FRP estimation from tropical peatlands.

We chose to investigate FRP in the tropical peatlands of the central Congo region because Dargie et al*.*^13^ discovered that the Congo Basin contains the most extensive tropical peatland complex in the world, most recently estimated by Crezee et al*.*^18^ to cover 167,600 km^2^, spanning both the Republic of the Congo (RoC) and Democratic Republic of the Congo (DRC). Our objectives were to estimate FRP for the peatlands of the central Congo Basin; compare them to nearby FRP from *terra firme* forest; assess if FRP alters with depth and season; and estimate the carbon fixed into fine roots on a per hectare basis. We hope this information will improve our understanding of the dynamics of the fine root carbon pool to provide information for biogeochemical modelling of peatland dynamics, the carbon cycle, and the impacts of future climate change on these ecosystems.

**Figure 1:**
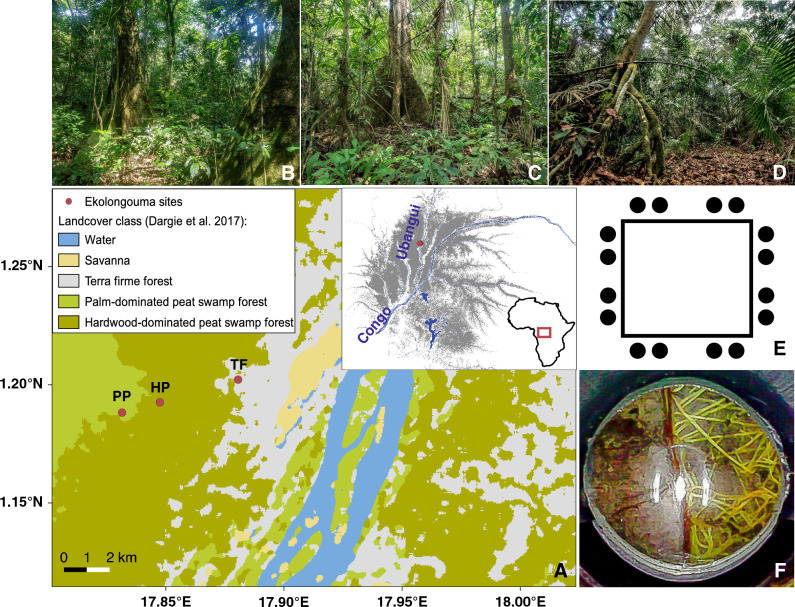
(**A**) Map of the study area in the Cuvette Centrale peatland complex (in dark grey) in the Congo Basin. Three inventory plots were installed in three ecosystem types (*terra firme*, hardwood-, and palm-dominated peat swamp forests). (**B–D**) Three pictures showing these environments, ordered as mentioned above. (**E**) A sketch of the location of the minirhizotron tubes installed in each inventory plot. (**F**) A picture taken using a minirhizotron system representing the root environment.

We installed the same system as Arnaud et al*.* installed in a waterlogged mangrove system^16^, in three adjacent sites in the Congo Basin: (i) a hardwood-dominated peat swamp forest, (ii) a palm-dominated peat swamp forest, and (iii) a *terra firme* tropical forest (Fig. 1). These are the two main peat swamp ecosystem types in the region and a control site to allow comparisons of our method with other *terra firme* tropical forests. We installed 16 tubes in each ecosystem type, placed systematically across a 100 × 100 m forest plot, each inserted to 1 m depth. Down-tube photographs were analysed at five depths (0–2.5 cm; 6–8.5 cm; 16–18.5 cm; 36–38.5 cm; and 71–73.5 cm, designed to capture rapid changes near the surface and corresponding to the depths 0–5, 5–10, 15–20, 35–40 and 70–75 cm) to estimate FRP growth over three one-month periods, May–June 2020, December 2020–January 2021, and February–March 2021, capturing the short wet season, long wet season, and long dry season, respectively (see “Methods and materials” section).

## Results

### Fine root production decreases with depth

**Figure 2:**
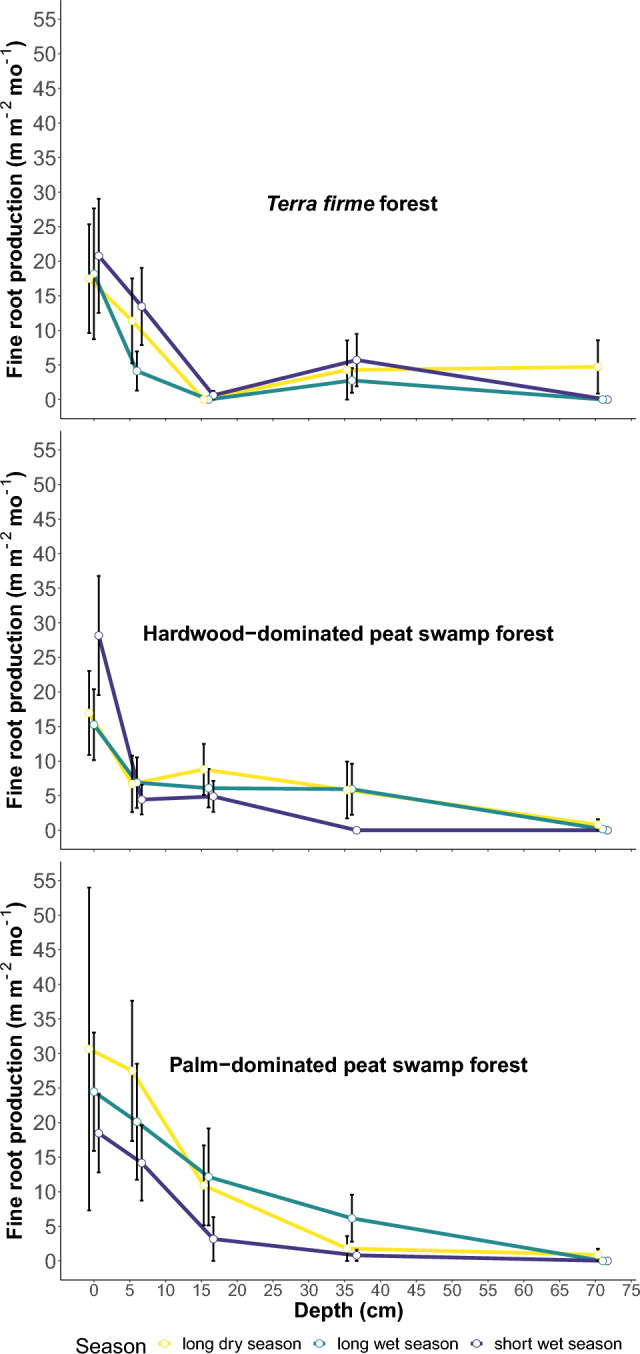
Measured marginal means and standard errors of fine root production monitored during three seasons per each ecosystem type, expressed in m m^−2^ mo^−1^ to a depth of 71 cm.

The minirhizotron data allow the estimation of new root production per image window area during a one-month interval between images (m m^-2^ mo^-1^). These measurements show a decline in FRP with depth across all three ecosystem types (Table 1; Fig. 2). Measured marginal means across all ecosystem types and seasons (16 tubes × 3 ecosystem types × 3 months) decreased from 21.2 ± 3.5 (± standard error (SE)) m m^-2^ mo^-1^ at 0–2.5 cm depth, to 12.1 ± 2.0 m m^−2^ mo^−1^ at 6–8.5 cm depth, 5.2 ± 1.2 m m^−2^ mo^−1^ at 16–18.5 cm depth, 3.7 ± 1.0 m m^−2^ mo^−1^ at 36–38.5 cm depth, and 0.7 ± 0.5 m m^−2^ mo^−1^ at 71–73.5 cm depth (Table 1). FRP is greatest in the shallowest depth interval (0–2.5 cm) in every forest type and season (Fig. 2 and Supplementary Table S1).

**Table 1:** Fine root production from *terra firme*, hardwood-dominated peat swamp, and palm-dominated peat swamp forests in the Congo Basin.

Depth (cm)	*Terra firme* mean (± SE)	Hardwood peat swamp mean (± SE)	Palm peat swamp mean (± SE)
0–2.5	18.82 (± 4.84)	20.14 (± 3.92)	24.53 (± 8.35)
6–8.5	9.66 (± 2.92)	6.01 (± 1.92)	20.59 (± 4.71)
16–18.5	0.21 (± 0.21)	6.60 (± 1.69)	8.75 (± 3.19)
36–38.5	4.26 (± 1.96)	3.92 (± 1.85)	2.92 (± 1.32)
71–73.5	1.57 (± 1.30)	0.32 (± 0.27)	0.29 (± 0.29)

Each value is the mean of 16 locations and three one-month measurements of root growth, expressed in m m^−2^ mo^−1^.

### Fine root production across the three ecosystem types

The comparison of the three ecosystem types shows that the *terra firme* and hardwood-dominated peat swamp forest have similar FRP, when averaged across the seasons and depth intervals and depth-weight corrected. *Terra firme* mean of 4.1 ± 1.7 (± SE) m m^−2^ mo^−1^ is similar to the mean from hardwood-dominated peat swamp forest at 4.7 ± 1.5 m m^−2^ mo^−1^ from 0 to 75 cm. Non-significantly higher FRP was found in the palm-dominated peat swamp forest where the rate was 6.6 ± 2.2 m m^−2^ mo^−1^ from 0 to 75 cm.

The pattern of the decrease in FRP with increasing depth varied between the *terra firme* and peat swamp ecosystem types (Table 1). In particular, the FRP mean dropped dramatically in the *terra firme* plot at a depth of 16–18.5 cm, while maintaining relatively high values in both the hardwood- and palm-dominated peatland sites. Thus, at 16–18.5 cm the terra firme forest had just 1.1% of the FRP found at 0–2.5 cm, compared to 32.8% and 35.7% in the hardwood- and palm-dominated peatland sites, respectively. A difference between the *terra firme* and the peat swamp forests was also evident at the deepest level (71–73.5 cm), where FRP was higher at the *terra firme*, being 8.3%, of the FRP at 0–2.5 cm, compared to just 1.6% and 1.2 % of the FRP at 0–2.5 cm in the hardwood-dominated and palm-dominated peat swamp sites, respectively. Overall, there were more surface roots and deeper roots, with few at intermediate depths in the *terra firme*, compared to the peat swamp forest ecosystems types.

### Fine root production and seasonality

When averaged across ecosystem types, depths intervals and depth-weight corrected, the measured marginal mean FRP reached the highest value during the driest month (February to March) at 6.2 ± 4.7 (± SE) m m^−2^ mo^−1^, when the water table dropped below the surface in the peat swamp sites. Non-significantly lower marginal means were observed during the short wet season (May to June) and the end of the long wet season (December–January), at 3.9 ± 2.5 m m^−2^ mo^−1^ and 5.4 ± 3.6 m m^−2^ mo^−1^, respectively. The *terra firme* site recorded the lowest FRP value during the large wet season at 2.5 ± 1.5 m m^−2^ mo^−1^. In addition, in the wet season virtually no FRP was seen at the greatest depth (71–73.5 cm) in all three ecosystem types. However, during the long dry season we observed differential seasonal redistribution of FRP across the soil profile and ecosystem types. During this season, a larger portion of FRP was allocated deeper in the soil (at a depth of 71–73.5 cm) in the *terra firme* site (27% of the FRP at 0–2.5 cm) compared to the hardwood-dominated and palm-dominated peat swamp forests (5% and 3% of the FRP at 0–2.5 cm, respectively, Fig. 2 and Supplementary Table S1).

### Modelling fine root production

We modelled FRP using a generalised linear mixed model, which we then analysed using an ANOVA. The ANOVA showed a significant effect of depth on FRP (p < 0.001, Table 2 for the ANOVA results, and Supplementary Table S2). The effect of ecosystem type was only significant in the interaction with depth (depth × ecosystem type), meaning that FRP is differently distributed across the soil profile in the three ecosystem types, but there was no significant difference in FRP for the main effect of ecosystem type. Similarly, the effect of season was only significant in interaction with depth (depth × season). This significant interaction indicates that FRP is allocated differently throughout the soil profile depending on the time of the year. During the short wet season, FRP declines dramatically with increasing depth in all three ecosystem types, whereas particularly in the driest month, February to March, a gentler decrease is observed, due to greater FRP allocation deeper in the profile (Supplementary Table S1 and Fig. S1). The main effect of season was not significant (see Table 2), though significantly higher FRP in the palm-dominated peat swamp forest, compared to the other vegetation types, was found at the end of the long wet season (December–January).

**Table 2:** Summary of ANOVA performed on fixed effects in a generalised linear mixed model**.**

Variable	Chi-squared	df	Significance
Intercept	56.124	1	< 0.001
Ecosystem type	1.427	2	0.478
Season	3.272	2	0.195
Depth	29.229	4	< 0.001
Ecosystem type × season	5.978	4	0.201
Season × depth	16.186	8	< 0.05
Ecosystem type × depth	28.674	8	< 0.001

### Fine root production dry mass estimates

We extrapolated FRP to 1 m depth in the soil profile, estimated it in dry mass, and converted this estimate to carbon units. First, we fitted a LOESS curve to the ANOVA means following the fitting of the generalised linear mixed model. FRP values were then predicted from this curve every 2.5 cm (corresponding to the minirhizotron observational window) up to 1 m depth, and then summed in intervals of 10 cm (Fig. 3). To convert to carbon we assume a depth of field (i.e., the depth into the exposed soil profile at which roots can be detected by the camera) of 2 mm^19^, specific root length (SRL) of 37.1, 45.1, and 51.6 m g^−1^^20^ for the depth intervals 0–10, 10–20, and 20–100 cm, respectively, and a root carbon content of 47.4%^21^ (see Table 3 and “Methods and materials”). Total FRP was 2.6 ± 0.3 (± SE), 1.9 ± 0.5, and 1.7 ± 0.1 Mg C ha^−1^ yr^−1^ at the palm-, hardwood-dominated peat swamp, and *terra firme* forest sites, respectively. Some 68.0–91.9% of FRP was found within the uppermost 30 cm of a 100-cm soil profile within each ecosystem type. Some 52.3–56.5% of the total FRP was found within the uppermost 10 cm in each ecosystem type. At deeper levels (30–70 cm), FRP was greater in the *terra firme* and hardwood-dominated peat swamp forest sites, in contrast with the palm-dominated peat swamp forest (~ 29.6%, 21.2% and 6.1% of the total FRP, respectively; Table 3, Fig. 3). At the deepest levels below 70 cm FRP was estimated close to zero (0.5 to 2.4%) in all three ecosystems (Table 3, Fig. 3).

**﻿Figure 3:**
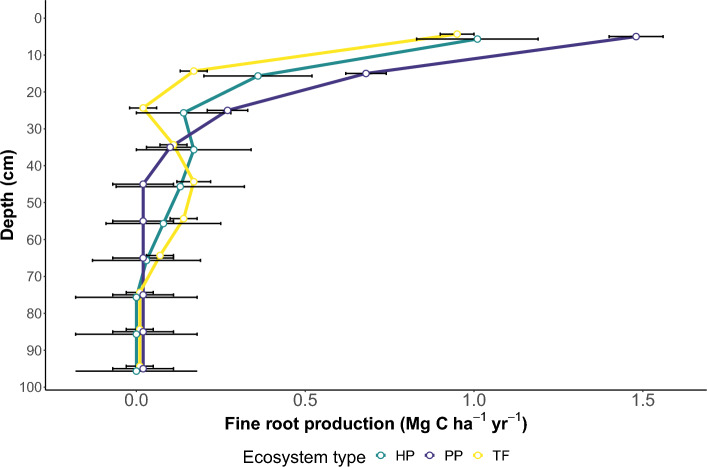
Mean fine root carbon production and uncertainty (SE), fitting a LOESS model to the ANOVA means from a generalised linear mixed model applied to measured values from five depths across a soil profile (0–71 cm), 16 locations and three months of root growth, for hardwood-dominated peat swamp forest (HP), palm-dominated peat swamp forest (PP) and *terra firme* forest (TF).

**Table 3:** Estimated fine root production from *terra firme*, hardwood-dominated peat swamp, and palm-dominated peat swamp forests in the Congo Basin.

Depth (cm)	*Terra firme* mean (± SE)	Hardwood peat swamp mean (± SE)	Palm peat swamp mean (± SE)
0–10	0.95 (± 0.05)	1.01 (± 0.18)	1.48 (± 0.08)
10–20	0.17 (± 0.04)	0.36 (± 0.16)	0.68 (± 0.06)
20–30	0.02 (± 0.04)	0.14 (± 0.14)	0.27 (± 0.06)
30–40	0.11 (± 0.04)	0.17 (± 0.17)	0.10 (± 0.07)
40–50	0.17 (± 0.05)	0.13 (± 0.19)	0.02 (± 0.09)
50–60	0.14 (± 0.04)	0.08 (± 0.17)	0.02 (± 0.09)
60–70	0.07 (± 0.04)	0.03 (± 0.16)	0.02 (± 0.09)
70–80	0.01 (± 0.04)	0.00 (± 0.18)	0.02 (± 0.09)
80–90	0.01 (± 0.04)	0.00 (± 0.18)	0.02 (± 0.09)
90–100	0.01 (± 0.04)	0.00 (± 0.18)	0.02 (± 0.09)
TOT	1.67 (± 0.13)	1.93 (± 0.54)	2.63 (± 0.27)

The values were extrapolated fitting a LOESS model to the ANOVA means of a generalised linear mixed model applied to measured values from five depths across a soil profile (0–71 cm), 16 locations and three months of root growth, in Mg C ha^−1^ yr^−1^.

## Discussion

We successfully deployed the recently-developed EnRoot minirhizotron system^16^ in palm-, hardwood-dominated peat swamp, and in *terra firme* forests in the world’s largest tropical peatland complex in the central Congo Basin. We estimated FRP for the palm- and hardwood-dominated peat swamp forests at 2.6 ± 0.3 (± SE) and 1.9 ± 0.5 Mg C ha^−1^ yr^−1^, down to a depth of 1 m. In addition, FRP in the *terra firme* forest in the Congo Basin, to a depth of 1 m, was 1.7 ± 0.1 Mg C ha^−1^ yr^−1^. Furthermore, to the best of our knowledge, this study is the first to compare FRP in peat and mineral soils from adjacent ecosystems. Our observational set-up and the minirhizotron method allowed us to observe subtle differences between the ecosystem types, rooting depths and seasons.

### Fine root production variation with depth

We found that around 68–92% of all new fine roots produced in one month at our forest sites were located in the uppermost 30 cm of the soil profile. This is in line with the few other reports of FRP in tropical forests. In an Amazon Basin *terra firme* forest, Cordeiro et al.^22^ found that around 60% of the total estimated FRP from a 1-m soil profile occurred in the 0–30 cm section, most of which occurred in the top 10 cm. Similarly, in a gallery forest (i.e., narrow patches of tropical forests that occur along the river valleys in savanna environments^23^) in the Congo Basin, Ifo et al.^24^ found that about 70% of total FRP was in the top 20 cm of a 40-cm soil profile. Although FRP was not reported, in an assessment of fine root stocks in three *terra firme* rainforests of Cameroon, Ibrahima et al.^25^ observed that 80% of fine root biomass was in the top layer (0–10 cm) of a 25-cm soil profile. Also, in a pre-montane evergreen rainforest in Sulawesi^26^ and in a hardwood-dominated peat swamp forest in Kalimantan^27^, Indonesia, similar fine root biomass depth distributions were reported, with about 75% in the uppermost 20 cm of soil across a 3 m soil profile and approximately 75–85% in the upper 0–25 cm of soil across a 50 cm section, respectively. The higher plant investment in FRP in the upper 30 cm of the soil profile is likely due to the higher concentration of nutrients in this zone compared to deeper layers^28^.

### Fine root production variation with seasonality

We observed seasonal changes in FRP with higher values during the driest periods of the year. In the *terra firme* forest these higher rates of FRP in the driest periods were deeper into the soil profile. Investment in FRP during the dry season may be a strategy used by canopy trees to cope with water deficit during drought periods^29^. Particularly, the allocation of FRP to deeper depths that we observed in the *terra firme* forest may be related to obtaining water in the driest part of the year, alongside abundant surface roots to obtain nutrients^28^, therefore resulting in low FRP at intermediate depths (Fig. 2). However, this observation is in contrast with other research in the tropics that found lower FRP during the dry season than in the wet season, with a slight increase in FRP in deeper layers during the latter season (Cordeiro et al.^22^ and references therein). We speculate that this differing pattern may be due to stronger water stress at our study site, where lower annual precipitation (~ 1700 mm vs. > 2000 mm) and stronger seasonality occur, compared to the cited study^22^.

### Carbon inputs from fine root production

Our estimated FRP of 1.7 ± 0.1 Mg C ha^−1^ yr^−1^ in the *terra firme* in the Congo Basin, down to a depth of 1 m, is comparable to FRP assessments from other locations in similar ecosystem types (Table 4). In an extensive review, Malhi et al.^30^ found that FRP ranged between 0.9 and 6.8 Mg C ha^−1^ yr^−1^ to a depth of 1 m, but this range did not include data from African tropical forests. More recently, at a series of *terra firme* sites in the western Congo Basin in Gabon, perhaps the most comparable to our *terra firme* sites, Huasco et al.^31^ estimated FRP comprised between 1.6 and 1.8 Mg C ha^−1^ yr^−1^ for a 30 cm profile using an ingrowth core, which is slightly higher than—although likely not significantly different from—the value in our study for the same depth interval (1.1 Mg C ha^−1^ yr^−1^). However, in a gallery forest in the central Congo Basin, Ifo et al.^24^ measured FRP to be 3.2 Mg C ha^−1^ yr^−1^ for a 0–40 cm soil profile using ingrowth cores.

**Table 4:** Fine root production (FRP) from this report and across the global tropics with reference, site of measurement, method used, and ecosystem type.

Reference	Site	Method	Depth (cm)	Ecosystem type	FRP (Mg C ha^−1^ yr^−1^)
Metcalfe et al. 2008^32^	Amazon Basin	i	0–30 cm	*Terra firme* tropical forest	1.4–3.3
Aragão et al. 2009^34^	Amazon Basin	i	0–100 cm	*Terra firme* tropical forest	2.2–7.6
Silver et al. 2005^33^	Amazon Basin	i	0–100 cm	*Terra firme* tropical forest	0.8–1.2
Cordeiro et al. 2020^22^	Amazon Basin	m	0–90 cm	*Terra firme* tropical forest	2.8
Hertel et al. 2009^26^	South-East Asia	i	0–100 cm	*Terra firme* tropical forest	0.9
Malhi et al. 2011^30^	Global tropics	i, r	0–100 cm	*Terra firme* tropical forest	0.9–6.8
Ifo et al. 2015^24^	Congo Basin	i	0–40 cm	Gallery forest	3.2
Huasco et al. 2021^31^	Congo Basin	i	0–30 cm	*Terra firme* tropical forest	1.6–1.8
This study	Congo Basin	m	0–100 cm	*Terra firme* tropical forest	1.7
Chimner and Ewel 2005^14^	Micronesia	i	0–30 cm	Tropical peat swamp forest	1.4
This study	Congo Basin	m	0–100 cm	Hardwood-dominated tropical peat swamp forest	1.9
This study	Congo Basin	m	0–100 cm	Palm-dominated tropical peat swamp forest	2.6

The abbreviations for the method used represent ingrowth core (i), minirhizotron (m), and rhizotron (r).

We found only one other study utilising a minirhizotron system (up to a depth of 90 cm) in a central Amazonian *terra firme* forest, which presents FRP values ~ 60% higher than the ones we report^22^, i.e. 2.8 Mg C ha^−1^ yr^−1^. However, comparable estimates to ours were reported at a *terra firme* site in the eastern Amazon Basin, ranging from 1.4–3.3 Mg C ha^−1^ yr^−1^, down to 30 cm^32^. Other studies show a wide range of FRP values in tropical *terra firme* forests, with FRP of rainforests in Amazonia being as low as 0.8 Mg C ha^−1^ yr^−1^^33^, to as high as 7.6 Mg C ha^−1^ yr^−1^^34^. Part of the variability in reported FRP may be due to methodological differences, from field methods to conversions to carbon content. Furthermore, climate, soil texture and nutrient content variation across sites may play an additional role in explaining this variability^31^. In summary, our FRP estimate for a Congo Basin *terra firme* forest is within the range of tropical forests worldwide (see Table 4), but it seems to be lower when compared to the range of FRP estimates from the Amazon Basin. However, it is unclear if FRP really is lower in African *terra firme* forests compared to Amazon *terra firme* forests, and if so, why. More data on FRP from tropical forests will be needed to elucidate any patterns and their causes.

We know of only one study of FRP in a tropical peat swamp forest, an ingrowth core study in a Micronesian tropical peatland, which reported a FRP of 1.4 Mg C ha^−1^ yr^−1^ down to 30 cm^14^. This value is very similar to our FRP estimate for a hardwood-dominated peat swamp forest—the most comparable of our sites to the Micronesia peatland—at 1.5 Mg C ha^-1^ yr^-1^ for a 30-cm section. On the other hand, this estimation for the hardwood-dominated peat swamp is only ~ 60% of our estimation at the palm-dominated site for a 30-cm section (2.4 Mg C ha^−1^ yr^−1^, see Table 3). Of the two peat swamp ecosystem types, the hardwood-dominated peat swamp forest site had lower fine root production (1.9 ± 0.5 Mg C ha^−1^ yr^−1^) than the palm-dominated peat swamp forest (2.6 ± 0.3 Mg C ha^−1^ yr^−1^) for a 1-m section, albeit non-significantly different (Table 2). The latter ecosystem type recorded the highest FRP among our sites, which is within the range found in *terra firme* forests worldwide (Table 4). Furthermore, the difference in FRP values recorded in the peatland forest sites compared to the *terra firme* was not significant. Nonetheless, future studies comparing FRP from adjacent, contrasting ecosystems types—as the one we report here—may shed further light on the role that FRP plays in accumulating organic material in tropical peat soils thus highlighting the importance of obtaining FRP measurements in such ecosystems.

## Limitations of the study

The key limitation of our study is that three-quarters of our individual measurements (i.e., for a particular combination of depth, ecosystem type, and month) showed no new live roots and so no root growth. This is likely because the sampling ‘window’ at each depth is small in each tube, with each photograph giving a depth of field into the soil of approximately 2 mm. This limited sampling appears to be the main drawback of using minirhizotrons compared to traditional ingrowth cores, which integrate a larger sampled volume per tube. We countered this limitation by sampling 16 locations across each ecosystem type over 1 ha, compared to the nine ingrowth cores per hectare recommended for non-swamp tropical forests^15^. Conversely, the advantages of using minirhizotrons over ingrowth cores are: (i) They avoid the much greater disturbance of the root environment caused by the deployment and retrieval of ingrowth cores, which can result in FRP underestimates. This lower disturbance is particularly important, as it is well-known that ingrowth cores underestimate root production, because roots must first grow through the disturbed soil and then the ingrowth chamber before they enter the measured volume and contribute to FRP^35^. (ii) Their ability to assess root growth deeper in the soil profile, with our minirhizotron tubes reaching 1 m depth compared to the typical 30-cm-deep ingrowth core^15^. (iii) The possibility of performing multiple repeat measurements once the minirhizotron is installed, allowing subtle seasonal changes in FRP to be more easily detected.

Overall, the conversion procedure, from root length units—derived from the digital minirhizotron photographs—to dry biomass estimates, has a number of uncertainties. We followed the method of Tingey et al.^36^, which first involves assuming a depth of field (see “Methods and materials”). The depth of field is used to estimate the volume of soil observed by the minirhizotron. The majority of research assume a depth of field of 2–3 mm^19^, though in one non-tropical study, it was measured as 1 mm^37^, and Taylor et al.^38^ suggest it could be lower than that. Here, we adopted a depth of field of 2 mm. However, this value is uncertain and has a large impact: with a depth of field of 1 mm, our FRP estimations would be among the high end of the range of FRP estimates from tropical forests^30^ (3–5 Mg C ha^−1^ yr^−1^). Future research to better define the depth of field from minirhizotrons is an important next step in obtaining more accurate FRP estimates from tropical forests. Secondly, to convert measurements from density of root length to root biomass density, we assume a specific root length (SRL), which is a measure of root length per unit mass^20^. We derived the SRL from Addo-Danso et al.^20^ (37.1, 45.1, and 51.6 m g^−1^ for the depth intervals 0–10, 10–20, and 20–100 cm, respectively), who determined it from 30-cm soil cores collected from a moist semi-deciduous forest on mineral soil in Ghana. To improve future minirhizotron FRP estimates for the central Congo Basin, future measurements of SRL from the *terra firme* and peatland forests are strongly needed.

Finally, our measurements covered a limited amount of time (three months), which includes some uncertainty when scaling to yearly estimates. Notwithstanding these limitations, we are able to provide the first estimates of FRP from the world’s largest peatland complex in the central Congo Basin that are comparable to estimated FRP in the tropics worldwide.

## Conclusion

In conclusion, we provide the first estimates of FRP from peat swamp and *terra firme* tropical forests in the Congo Basin to a depth of 1 m. Our FRP estimation in a *terra firme* forest is comparable to selected sites in the Amazon, central Africa, and South-East Asia. In the two peat swamp ecosystem types, the palm-dominated peat swamp forest recorded higher FRP than the hardwood-dominated peat swamp forest site, with the latter estimate being close to the only previous assessment in a hardwood-dominated tropical peat swamp forest, in Micronesia. In addition, while we recorded higher FRP values in the peatland forest sites compared to the *terra firme*, this difference was not significant. Our results show that it is possible to measure FRP in inundated peatlands using the EnRoot minirhizotron^16^, which is able to reveal subtle changes, such as greater fine root production at the greatest depths in the soil profile in the driest month of the year. We suggest that this method should be used to measure FRP in tropical environments, especially in wetlands. However, measurements of the (i) specific root length and (ii) depth of field of the minirhizotron are strongly necessary to more accurately convert the root length estimates from the minirhizotron photos to units of dry mass per volume of soil.

## Methods and materials

We used the EnRoot minirhizotron method^16^ to estimate FRP in three different types of primary moist forests of the central Congo Basin: a *terra firme* forest, and hardwood- and palm-dominated peat swamp forests. The measurements were made between May 2020 and March 2021. Permission to undertake the study was given by the government of the Republic of the Congo. No voucher specimens were collected during the field campaigns.

### Study area

This study was conducted in the Cuvette Centrale, an area in the Congo Basin rainforest biome characterised by primary tropical moist forest that forms extensive peatlands (mean estimated peatland area: 167,600 km^2^, mean estimated thickness 1.7 m^18^. Our study site was located in the Likouala district, Republic of the Congo. The annual temperature is 25.6 °C, and rainfall is approximately 1700 mm year^−1^, characterised by a bimodal regime with two peaks between March to May and September to November^39^. Three long-term 1 ha forest inventory plots were installed in January to March 2019 by the CongoPeat project (https://congopeat.net) in three different ecosystem types (Fig. 1, Supplementary Table S4): (i) *terra firme* forest, (ii) hardwood-dominated peat swamp forest, and (iii) palm-dominated peat swamp forest.

The *terra firme* plot is characterised by closed canopy-forming species (mean tree height: 26.5 m, personal observation) such as *Angylocalyx* sp. Taubert, *Strombosiopsis tetrandra* Engl. & Prantl, and *Symphonia globulifera* L.f. The number of trees with diameter larger than 10 cm at breast height was 378 ha^−1^, while the basal area of all trees in the plot was 25.3 m^2^ ha^−1^ in 2020–2021.

The hardwood and palm peat swamp forest plots were installed in forested peatlands and both sites have the water table at or above the peat surface for most of the year. The hardwood peat swamp forest contains peat to a maximum depth of 1.8 m; minimum and maximum water table levels at this site are − 0.60 m (subsurface) and 0.22 m (ponding), respectively^40^. This ecosystem type is characterised by the dominance of high-stem, hardwood species such as *Carapa procera* DC., *Grossera macrantha* Pax, and *Uapaca paludosa* Aubrév. & Leandri, a relatively high (mean tree height: 18.2 m, personal observation) and closed canopy. The number of trees with diameter larger than 10 cm at breast height was 382 ha^−1^ and the basal area of all trees in the plot was 29.7 m^2^ ha^−1^ in 2020–2021.

The palm-dominated peat swamp forest has a maximum peat depth of 3.0 m; minimum and maximum water-table levels are recorded between 0.14 m below and 0.24 m above the ground surface, respectively^40^. This forest is dominated by the palm *Raphia laurentii* De Wild., which has a low stature (~ 9 m maximum palm height, personal observation), and a non-continuous tree canopy with mean tree height of 17.6 m (personal observation). The numbers of trees having a diameter larger than 10 cm at breast height was 85 ha^−1^ and the basal area of all trees in the plot was 7.6 m^2^ ha^-1^ in 2020–2021.

### Measurement set-up

The Global Ecosystem Monitoring (GEM) network^15^ provides the most widely used protocols for carbon monitoring in the tropics. FRP is monitored by the GEM network using ingrowth cores or rhizotrons. The former consists of a cylinder made of a plastic mesh installed into the soil filled with root-free soil. Though frequently used in *terra firme* environments^11, 41, 42^, the GEM ingrowth core method is not effective in tropical peatlands for the following reasons. The manipulation of the medium for the experimental purpose—removal of roots to retain soil to fill the cores—is hindered by waterlogged conditions for a large part of the year. Furthermore, due to the intricate root mat that permeates the superficial layer of peatlands, it is difficult, if not impossible, to remove all roots from the peat. Critically, the GEM protocol for obtaining root-free soil for use in ingrowth cores is cumbersome to use in peats, and not all roots can be extracted. These residual roots may be counted as new roots after core extraction, leading to an over-estimation of FRP. Rhizotrons are also used in GEM and comprise a plastic window installed in a shallow soil pit^15^. They are not suitable for peatlands, where the soil is not stable, and where groundwater inundation makes measurements (photographs through the window) impossible for most of the year.

Considering these limitations, we installed a new minirhizotron system, EnRoot, that was previously successfully applied in mangrove environments^16, 17^. The EnRoot minirhizotron consists of two main components: a soil tube and an imaging system^16^. The soil tube is a 2-m-long, transparent cylinder with an external diameter of 32 mm, permanently installed in the soil to a depth of 1 m; while the imaging system consists of a camera apparatus, and an indexing handle that allows the camera to be raised, lowered and rotated for repeat photography with consistent positioning. The soil tube and camera system were custom made at the School of Geography, University of Leeds, following the exact specifications in Arnaud et al.^16^. Sixteen soil tubes were installed in each of the three ecosystem types (to account for heterogeneity), in couples at each corner of each study plot (at a distance of more than 1 m, see Fig. 1) on the 18th, 19th, and 21st of January 2020 for the palm-dominated peat swamp forest, hardwood-dominated peat swamp forest, and *terra firme* forest, respectively, for a total of 48 tubes.

The tubes were installed vertically due to the dense root mat that characterises the peat soils. After letting the soil around the tubes settle for four months to reduce any potential impacts of disturbance after installation, monthly measurements were carried out to capture FRP over one-month periods from 17–19/05/2020 to 21–26/06/2020 (mid-short wet season; water table close to the surface), 15–21/12/2020 to 20–25/01/2021 (end long wet season; water table above the surface), and 18–21/02/2021 to 17–27/03/2021 (peak long dry season; water table well below the surface). These time intervals were chosen to minimise the number of roots appearing and dying within the census interval, which would not be measured and thus lead to loss of information.

The imaging system is inserted into the soil tube, allowing the user to take images of the root environment at different depths and orientations via the simple use of a smartphone connected to the camera apparatus. Given the number of photographs that could be produced and time to analyse each, we only took photographs at selected depths. We chose 5 depths covering part of the following depth ranges 0–5, 5–10, 10–20, 20–40, and 40–80 cm. The focus of our measurements on the first 40 cm of soil was based on the observation from the literature that roots tend to concentrate in this depth interval^21, 25, 26^. Photographs of the soil were taken at five depths: 0–2.5, 6–8.5, 16–18.5, 36–38.5, 71–73.5 cm, as the photos cover 2.5 cm of depth in the tubes. At each depth, ten partially overlaying pictures were taken at different orientations, corresponding to a 360° view of the root environment. Consecutive monthly images were then analysed to measure the addition of root length over time (reported as m of new roots per m^2^ area of photo window per month), then converted into biomass carbon, as Mg C ha^−1^ yr^−1^ (see next section) to determine FRP in units comparable to other studies and for future integration into ecosystem carbon stock and flow studies (sensu Malhi^6^).

### FRP measurements, calculations, and statistical analyses

RStudio was used to carry out the statistical analysis reported here^43^. We analysed pictures from 16 minirhizotron tubes for each month of data collection for each ecosystem type. We randomly picked one orientation for the five depths covered by our data collection for three one-month periods. A total of 1440 minirhizotron images (3 ecosystem types × 16 tubes × 5 depths × 6 census dates) were first digitally corrected for distortion (due to the cylindrical soil tube and the camera lens) assigning calibrated coordinates to the distorted images in QGIS^44^. Next, the images were flattened using GDAL^45^ in Python, utilising the script developed by Arnaud et al.^16^. Image quality was then improved employing Adobe Lightroom. Lastly, changes in root length between consecutive pairs of images were analysed operating rhizoTrak, open-source software designed to analyse root length in minirhizotron images^46^, see Fig. 1. FRP was expressed as m m^−2^ mo^−1^ for each ecosystem type and each depth sampled.

A generalised linear mixed model (GLMM) was used to test the effect of three categorical predictor variables (*ecosystem type*, *depth*, and *season*), and their two-way interactions (*ecosystem type* × *depth*, *ecosystem type* × *season*, *depth* × *season*), on our FRP values from each of the 16 tubes per ecosystem type. We specified the models using the glmmTMB function from the R package GLMMTMB^47^ because FRP is zero-inflated and so highly positively skewed (at specific tubes, depths, and time no roots and thus no root growth are recorded). To account for the zero-inflated response variable we selected the Tweedie distribution (a log link function) as a family function, following Arnaud et al*.*^17^. We included a random intercept to account for potential unequal variance arising from hierarchical clustering of measurements within coring locations. We also included random intercepts for each month, to account for any temporal changes between our repeated measures. Akaike’s Corrected Information Criterion (AICc) analysis was conducted to evaluate which variables to include in the model. The inclusion of random intercepts for individual tubes and months led to a highly non-significant increase in model performance according to change in AICc compared to a model that omitted the random intercepts (p = 0.423). Nonetheless, we chose to retain the random intercept for individual tubes in the final model in the interests of caution. The deviation between observed and expected residuals was not significant. Furthermore, no significant problems were detected between the standardised residuals and the (rank transformed) model predictions.

Once we used the AICc to specify the model’s variables, we analysed our GLMM using the ANOVA function from the R package CAR^48^. We used a Type-III sum-of-squares to analyse the significance of the main effects and their interaction based on Chi-Square tests of their fitted values. We used the emmeans function from the R package EMMEANS^49^ to conduct Tukey post hoc comparisons between estimated marginal means for all pairs of samples.

### Conversion of FRP to carbon units

To extrapolate values for a 1-m soil profile from our minirhizotron-derived FRP data at five depths, we fitted a LOESS model to the ANOVA means derived from the GLMM for each ecosystem type, season, and depth. From the fitted curve, we predicted FRP values every 2.5-cm interval to a depth of 1 m. Successively, FRP values within a 10-cm section were summed up. Overall SE was calculated as the square root of the sum of the squared values of each individual depth measurement SE. This procedure allowed us to estimate FRP from 0–100 cm depth in units of m m^−2^ mo^−1^.

We converted these FRP values, expressed as length changes through time, into carbon content units, following Tingey et al*.*^36^ (see Supplementary Table S5). First, we multiplied the surface of the window frame by a depth of field of 2 mm^19^, to estimate the sampled volume by the minirhizotron. The roots observed in the minirhizotron frame are assumed to fill some soil volume, and the depth of field is a recommended estimate that is assigned to calculate this volume^36^. Secondly, the density of root length (m m^−3^) was calculated by dividing the length of fine root (m yr^−1^) in each video frame by the soil volume observed, and then divided by an estimated specific root length (SRL, m g^−1^) to obtain root biomass density (g m^−3^). The SRL is the length per unit mass^36, 50^. Given the lack of SRL estimates at our sample location, we used an SRL of 37.1, 45.1, and 51.6 m g^−1^, for the depth intervals 0–10, 10–20 and 20–100 cm, respectively, as calculated by a study at a moist semi-deciduous forest in Ghana using root dry mass from ingrowth cores^20^. Root biomass density was then scaled to biomass per surface area (g m^−2^) by multiplying it by the depth of the soil profile (10 cm per section), and lastly converted into Mg C ha^−1^ yr^−1^ units, assuming a carbon content of 47.4%^21^ and multiplying the monthly estimate by twelve.

### Ethical standards

The research was carried out within the guidelines and legislation of the Republic of the Congo where fieldwork took place. All root samples were processed in the Republic of the Congo.

## Supplementary Information

Supplementary Information.

## Data Availability

Data will be uploaded on the CongoPeat project website (https://congopeat.net) upon acceptance. Minirhizotron photos of the root monitoring are available upon request from the corresponding author.
