# Supplementary material for: First estimates of fine root production in tropical peat swamp and terra firme forests of the central Congo Basin
Source: Sci Rep. 2023 Jul 29;13:12315. doi: 10.1038/s41598-023-38409-x (PMC10387053; doi:10.1038/s41598-023-38409-x)
Supplement: Supplementary file 1 — Supplementary Information. [file 41598_2023_38409_MOESM1_ESM.docx]

Supplementary information:

First estimates of fine root production in tropical peat swamp and *terra firme* forests of the central Congo Basin.

Sciumbata et al.

**Supplementary Table S1.** Estimated mean  and standard error (SE) of fine root production (FRP) for minirhizotron measurements in three ecosystem types (*terra firme*, hardwood-, and palm-dominated peat swamp forest) and for three seasons in the Congo Basin. Values are expressed as m m^-2^ mo^-1^. N = 16.

| **month** | **season** | **depth** |  | **Ecosystem type** |  |
| --- | --- | --- | --- | --- | --- |
|  |  | **(cm)** | ***terra firme*** | **hardwood peatland** | **palm peatland** |
| **May–June 2020** | small wet season | 0–2.5 | 20.79 (± 8.26) | 28.17 (± 8.62) | 18.48 (± 5.67) |
| **May–June 2020** | small wet season | 6–8.5 | 13.46 (± 5.58) | 4.44 (± 2.13) | 14.17 (± 5.43) |
| **May–June 2020** | small wet season | 16–18.5 | 0.62 (± 0.62) | 4.89 (± 2.24) | 3.17 (± 3.17) |
| **May–June 2020** | small wet season | 36–38.5 | 5.72 (± 3.78) | 0.00 (± 0.00) | 0.80 (± 0.80) |
| **May–June 2020** | small wet season | 71–73.5 | 0.00 (± 0.00) | 0.00 (± 0.00) | 0.00 (± 0.00) |
| **December 2020–January 2021** | end long wet season | 0–2.5 | 18.18 (± 9.47) | 15.27 (± 5.13) | 24.47 (± 8.55) |
| **December 2020–January 2021** | end long wet season | 6–8.5 | 4.13 (± 2.84) | 6.89 (± 3.66) | 20.12 (± 8.40) |
| **December 2020–January 2021** | end long wet season | 16–18.5 | 0.00 (± 0.00) | 6.09 (± 2.78) | 12.15 (± 7.01) |
| **December 2020–January 2021** | end long wet season | 36–38.5 | 2.77 (± 1.79) | 5.94 (± 3.69) | 6.16 (± 3.40) |
| **December 2020–January 2021** | end long wet season | 71–73.5 | 0.00 (± 0.00) | 0.16 (± 0.16) | 0.00 (± 0.00) |
| **February–March 2021** | end long dry season | 0–2.5 | 17.48 (± 7.86) | 16.97 (± 6.08) | 30.65 (± 23.35) |
| **February–March 2021** | end long dry season | 6–8.5 | 11.39 (± 6.13) | 6.69 (± 4.07) | 27.48 (± 10.14) |
| **February–March 2021** | end long dry season | 16–18.5 | 0.00 (± 0.00) | 8.81 (± 3.69) | 10.92 (± 5.79) |
| **February–March 2021** | end long dry season | 36–38.5 | 4.28 (± 4.28) | 5.82 (± 4.11) | 1.79 (± 1.79) |
| **February–March 2021** | end long dry season | 71–73.5 | 4.72 (± 3.86) | 0.80 (± 0.80) | 0.88 (± 0.88) |

Supplementary table S2.  Summary of random and fixed effects for our generalised linear mixed model. Since the intercept is a constant it has no levels. The reference categories have a zero estimate. Model’s formula: fine root production ~ (ecosystem type + season + depth + (ecosystem type × season) + (depth × season) + (depth × plot) + (1|sample) + (1|season)); AIC = 2000.7. Number of observations: 720, variables: sample, 16; season, 3.

| random variable | estimate | std. deviation |
| --- | --- | --- |
| sample | 0.07198 | 0.2683 |
| season | < 0.001 | < 0.001 |

| fixed variable | level | estimate | std. error | z | p |
| --- | --- | --- | --- | --- | --- |
| intercept | – | 2.539 | 0.3390 | 7.492 | < 0.001 |
| ecosystem type | *terra firme* | 0 | – | – | – |
|  | hardwood-mixed peatland | -0.3115 | 0.4415 | -0.705 | 0.480501 |
|  | palm-dominated peatland | -0.5417 | 0.4476 | -1.210 | 0.226202 |
| season | short wet season | 0 | – | – | – |
|  | end long wet season | -0.8142 | 0.4637 | -1.756 | 0.079144 |
|  | end long dry season | -0.5378 | 0.4505 | -1.194 | 0.232560 |
| depth (cm) | 0–2.5 | 0 | – | – | – |
|  | 6–8.5 | -0.8177 | 0.4573 | -1.788 | 0.073734 |
|  | 16–18.5 | -4.960 | 1.115 | -4.450 | < 0.001 |
|  | 36–38.5 | -2.158 | 0.6035 | -3.577 | 0. 000348 |
|  | 71–73.5 | -29.01 | 4418 | -0.007 | 0.994761 |

**Supplementary Figure S1.** Mean fine root carbon production and uncertainty (standard error, SE), estimated fitting a LOESS model to the ANOVA means from a generalised linear mixed model applied to measured values from five depths across a soil profile (0–71 cm), 16 locations, for *terra firme* forest (TF), hardwood-dominated peat swamp forest (HP) and palm-dominated peat swamp forest and three monitored seasons.


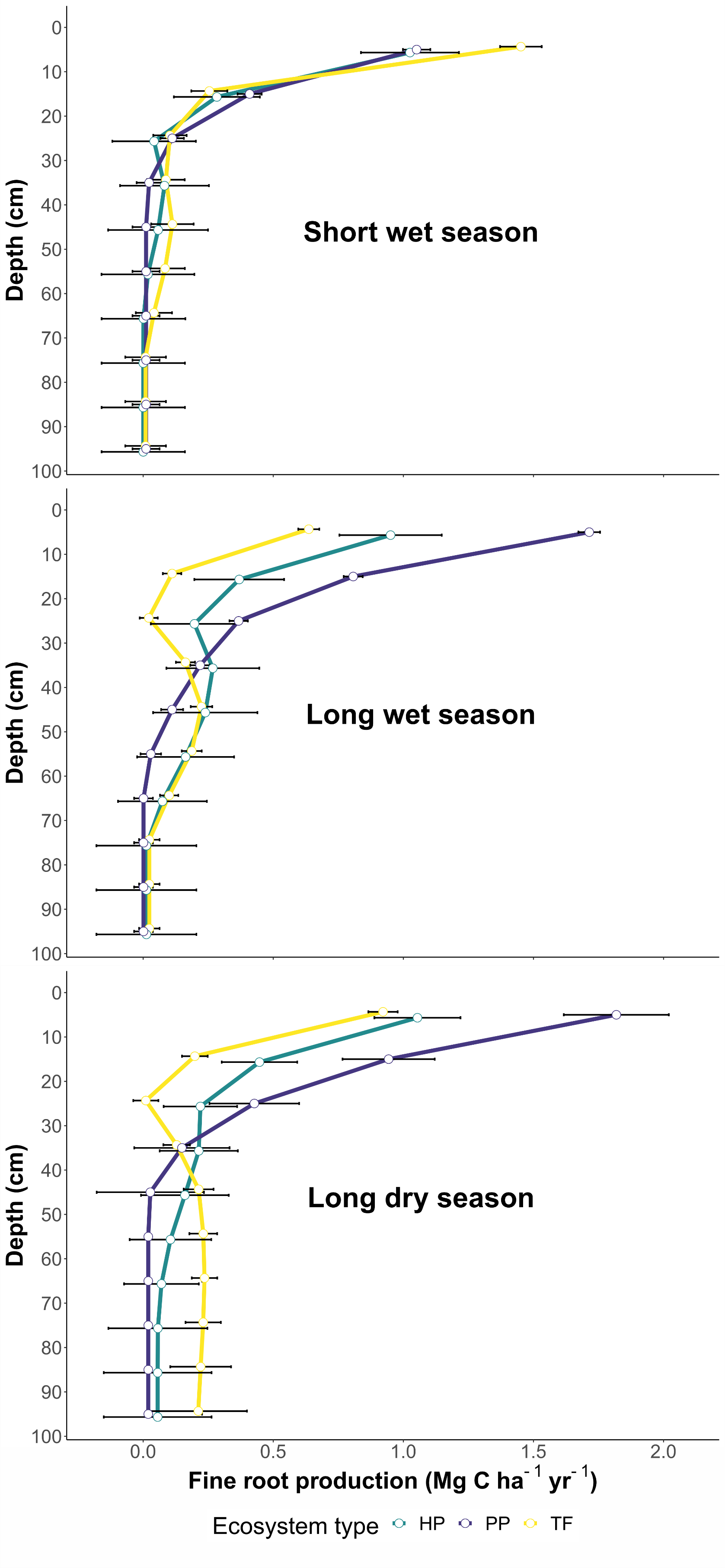


**Supplementary Table S3.** Fine root production from *terra firme* forest, hardwood-dominated peat swamp forest, and palm-dominated peat swamp forest in the Congo Basin. Each value is the mean of 16 locations expressed in Mg C ha^-1^ yr^-1^.

| **month** | **season** | **depth** |  | **Ecosystem type** |  |
| --- | --- | --- | --- | --- | --- |
|  |  | **(cm)** | ***terra firme*** | **hardwood peatland** | **palm peatland** |
| **May–June 2020** | small wet season | 0–30 | 1.81 (± 0.12) | 1.35 (± 0.29) | 1.57 (± 0.08) |
| **May–June 2020** | small wet season | 30–60 | 0.28 (± 0.13) | 0.16 (± 0.31) | 0.04 (± 0.09) |
| **May–June 2020** | small wet season | 60–100 | 0.07 (± 0.15) | 0.00 (± 0.32) | 0.04 (± 0.10) |
| **May–June 2020** | small wet season | TOT | 2.16 (± 0.26) | 1.51 (± 0.59) | 1.66 (± 0.18) |
| **December 2020–January 2021** | end long wet season | 0–30 | 0.77 (± 0.06) | 1.52 (± 0.31) | 2.89 (± 0.06) |
| **December 2020–January 2021** | end long wet season | 30–60 | 0.57 (± 0.07) | 0.67 (± 0.33) | 0.36 (± 0.07) |
| **December 2020–January 2021** | end long wet season | 60–100 | 0.17 (± 0.08) | 0.11 (± 0.37) | 0.00 (± 0.07) |
| **December 2020–January 2021** | end long wet season | TOT | 1.51 (± 0.13) | 2.30 (± 0.65) | 3.25 (± 0.13) |
| **February–March 2021** | end long dry season | 0–30 | 1.13 (± 0.09) | 1.72 (± 0.26) | 3.19 (± 0.32) |
| **February–March 2021** | end long dry season | 30–60 | 0.57 (± 0.09) | 0.48 (± 0.28) | 0.20 (± 0.34) |
| **February–March 2021** | end long dry season | 60–100 | 0.90 (± 0.24) | 0.24 (± 0.38) | 0.08 (± 0.41) |
| **February–March 2021** | end long dry season | TOT | 2.60 (± 0.31) | 2.44 (± 0.60) | 3.46 (± 0.70) |

**Supplementary Table S4.** Coordinates of the three long-term 1 ha forest inventory plots installed in three different ecosystem types in the Republic of the Congo where the study was conducted.

| **ecosystem type** | **latitude** | **longitude** |
| --- | --- | --- |
| *terra firme* forest | N1.20233 | E17.88064 |
| hardwood-dominated peat swamp forest | N1.191998125 | E17.846925 |
| Palm-dominated peat swamp forest | N1.18828 | E17.83152 |

**Supplementary Table S5.** Illustration of the procedure used to convert fine root production estimates from m m^-2^ mo^-1^ to Mg C ha^-1^ yr^-1^ units, following Tingey *et al.*^38^, see “Conversion of FRP to carbon units” section in Methods and Materials for a detailed explanation of the procedure.

|  | minirhizotron sampled volume (m^3^) = minirhizotron window frame (m^2^) * depth of field (m) |
| --- | --- |
|  | density of root length (m m^-3^ yr^-1^) = fine root length (m yr^-1^) / minirhizotron sampled volume (m^3^) |
|  | root biomass density (g m^-3^ yr^-1^) = density of root length (m m^-3^ yr^-1^) / specific root length (m g^-1^) |
|  | biomass per surface area (g m^-2^ yr^-1^) = root biomass density (g m^-3^ yr^-1^) * depth of soil profile (m) |
|  | biomass in C units (Mg C ha^-1^ yr^-1^ ) = biomass per surface area (Mg ha^-1^ yr^-1^) * carbon content (C * g^-1^) |
